# Social Requests and Social Affordances: How They Affect the Kinematics of Motor Sequences during Interactions between Conspecifics

**DOI:** 10.1371/journal.pone.0015855

**Published:** 2011-01-24

**Authors:** Francesca Ferri, Giovanna Cristina Campione, Riccardo Dalla Volta, Claudia Gianelli, Maurizio Gentilucci

**Affiliations:** 1 Department of Neuroscience, University of Parma, Parma, Italy; 2 Brain Center for Social and Motor Cognition, Italian Institute of Technology, Parma, Italy; Royal Holloway, University of London, United Kingdom

## Abstract

The present study aimed at determining whether and what factors affect the control of motor sequences related to interactions between conspecifics. Experiment 1 demonstrated that during interactions between conspecifics guided by the social intention of feeding, a social affordance was activated, which modified the kinematics of sequences constituted by reaching-grasping and placing. This was relative to the same sequence directed to an inanimate target. Experiments 2 and 4 suggested that the related-to-feeding social request emitted by the receiver (i.e. the request gesture of mouth opening) is prerequisite in order to activate a social affordance. Specifically, the two experiments showed that the social request to be fed activated a social affordance even when the sequences directed towards a conspecific were not finalized to feed. Experiment 3 showed that moving inside the peripersonal space of a conspecific, who did not produce any social request, marginally affected the sequence. Finally, experiments 5 and 6 indicated that the gaze of a conspecific is necessary to make a social request effective at activating a social affordance. Summing up, the results of the present study suggest that the control of motor sequences can be changed by the interaction between giver and receiver: the interaction is characterized by a social affordance that the giver activates on the basis of social requests produced by the receiver. The gaze of the receiver is a prerequisite to make a social request effective.

## Introduction

According to the Fitts' law [1] the duration of movements directed to a target lengthens, and in general the kinematics of transitive actions (i.e. acted upon an object, [2]–[5]) slow down, with an increasing index of movement difficulty. The index of difficulty is directly proportional to movement amplitude and inversely proportional to target size. Increasing movement difficulty induces greater accuracy during movement execution. However, other factors can affect accuracy during action control. Marteniuk et al. [4] found that the final phase of the reaching-grasping of an object lengthened when the successive movement was placing it into a container, as compared to less accurate movements, like throwing it. The data of other kinematic studies [6]–[8] confirmed that the overall intention of an action sequence could induce changes in the kinematics of even the initial actions. In other words, the overall intention can influence the degree of accuracy of each action of a sequence. These findings are in accordance with data of single neuron recording studies: Fogassi, Ferrari, Gesierich, Rozzi, Chersi and Rizzolatti [9] recorded neurons in monkey parietal cortex that discharged when the animal executed the grasping of a piece of food in order to bring it to its mouth. In contrast, they did not discharge when the second action was placing it into a container beside the monkey's mouth. The authors proposed that these neurons code the overall intention of the sequence (i.e. they code “why” an object is grasped).

The above cited studies [2]–[8] indicate that the affordances of an object, i.e. the types and motor patterns of interaction with an object (for a review see [10]), also depend on the final intention of actions, and, broadly speaking, on the context in which the actions are executed. On the basis of this idea, Loveland [11] proposed other two types of affordances: the culturally selected affordances and the social affordances. The culturally selected affordances reflect preferred but not necessary interactions. They are due to participation with other people in a shared cultural milieu that predisposes individuals to use objects in particular ways. The social affordances reflect the meaning of human activity, like for example request gesture, which indicates to other individuals a required type and pattern of interaction. The activation of social affordances is typical of interactions between conspecifics.

Becchio, Sartori, Bulgheroni and Castiello [12] reported that interacting with a conspecific by a sequence constituted by the actions of reaching-grasping an object and placing it on the hand of a conspecific induces variation in the kinematics of the actions, as compared to the same sequence directed to a container. Specifically, during the reach-to-grasp action they observed a decrease in the maximal finger aperture and peak grip closing velocity when interacting with the conspecific. The authors attributed these effects to the social affordance of the sequence, since placing an object in the conspecific's hand is performed in order to “give” and is characteristic of joint actions. Ferri, Campione, Dalla Volta, Gianelli and Gentilucci [13] found that when a giver reaches to grasp and places a piece of food into the mouth of a human receiver in order to feed her, the final phase of reaching and the placing slow down. This was relative to the execution of the same sequence directed to a mouth-like aperture on the “face” of a human body shape (non-human receiver). In this study the interaction with a conspecific likely activated a social affordance, too.

The results of these studies [12], [13] suggest that the social affordances guiding approaching action sequences increases the accuracy demand during the execution of these sequences. Alternatively, the increasing demand of accuracy may be explained by the fact that such actions are executed inside the conspecific's peripersonal space, where the probability of touching the receiver's body is higher [14]. In addition, if actions approaching a conspecific activate social affordances that increase accuracy demand, the question arises if and how the corresponding social requests (i.e. the request gestures) play a role in activating social affordances and consequently in modifying the kinematics of the sequences.

We addressed these problems in the present kinematic study through six experiments in which we compared sequences guided by social affordances related to approaching a conspecific (human receiver), with sequences guided by affordances related to approaching an inanimate target (non-human receiver). In baseline experiment 1, participants (the givers) reached-grasped a sugar lump and placed it into either the mouth of a conspecific (i.e. fed her) or a mouth-like aperture in a human body shape (i.e. placed it). Distances and size of the two final targets, i.e. their indices of difficulty according to the Fitts' law [1], were the same. Consequently, if the interaction with conspecifics increases the accuracy demand, a specific social affordance is likely activated, and the kinematics of reaching-grasping as well as of placing should be slowed down. If the hypothesis is incorrect, no slowing down of movement should be observed. In experiments 2, 3 and 4 we tested the role of the peripersonal space in affecting the accuracy requirements of the sequence (experiment 3) and the role of the social request to be fed (experiments 2 and 4) in activating the corresponding social affordances. Specifically, in experiments 2 and 4, we verified whether a social affordance is activated by the social request to be fed even when the sequence is directed to the conspecific in order to place (without any direct interaction with the conspecific, experiment 2) and to touch (experiment 4) rather than to feed.

Previous studies showed that during social interactions the receiver's gaze can be a signal in order to initiate a communication and even a joint action [15]–[17]. Thus, we aimed at verifying whether the receiver's gaze plays a role in making a social request effective at activating a social affordance (experiments 5 and 6).

## Experiment 1

**Figure 1:**
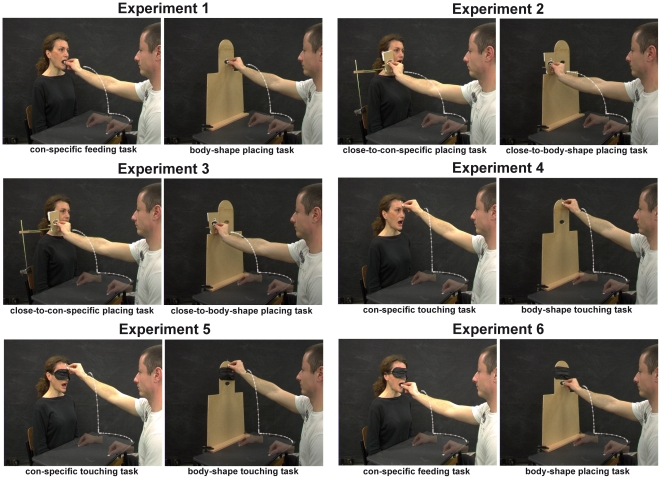
Experimental set-up, stimuli and examples of the action sequences performed by the participants in experiments 1–6. White lines represent examples of wrist trajectories. The actor and participant have seen this manuscript and figure and have provided written consent for publication.

In experiment 1, we tested whether and to what extent the interaction with a conspecific guided by the social intention of feeding modifies the kinematics of a sequence constituted by reaching-grasping and placing. In other words, we aimed at verifying whether a social affordance was activated. The activation of a social affordance can concern the interaction either with a specific part of the conspecific's body (in the present experiment, the mouth) or with the entire conspecific's body. Indeed, in the present experiment, the reaching and the initial placing were dire cted towards the conspecific's chest (Fig. 1). If the first hypothesis is correct the slowing down of movement should produce a decrease in the variability of the placing end point, since the givers reduced the effective target width (size disposable to introduce the food into the mouth) in order to avoid touching the receiver's lips. If the second hypothesis is correct, a decrease in the variability in the placing end point is unlikely to be found.

### Methods

#### Participants

Twelve right-handed [18], naïve volunteers (4 females and 8 males, age 22–25 yrs.) participated in the experiment. The Ethics Committee of the Medical Faculty at the University of Parma approved the study. The experiments were conducted according to the principles expressed in the Declaration of Helsinki. We obtained written informed consent from all participants in the present study.

#### Apparatus and stimuli

The participants (the givers) sat in front of a table on which they placed their right hand with the thumb and index finger in pinch position (Starting Position, SP). Depending upon the task condition, either an experimenter (the human receiver) sat, or a human body shape (the non-human receiver) was placed in front of them. The same female experimenter participated as human receiver in all experiments. The human receiver's chest, or the body shape, was 38 cm distant from the SP. The body shape was a wooden panel, the outline of which resembled the head, and the upper trunk of a human body. The “face” of the human-like shape had an ellipse-shaped slit resembling a mouth (mouth-like aperture). The size of this mouth-like aperture was approximately the same as that of the human receiver's mouth (when it was opened) and the distance of its center from the table plane (42.5 cm) was equivalent to that for the human mouth. Behind the mouth-like aperture, a support allowed an easy placing of a small object. The target of the reach-grasp action (see below) was a sugar lump (cube of 1×1×1cm) placed on the table plane in front of the participant at a distance of 16 cm from SP.

#### Procedure

The participants (the givers) executed the following two tasks depending on whether either the conspecific (i.e. the human receiver) or the body shape (i.e. the non-human receiver) was present: 1) reaching-grasping and placing the sugar lump into the conspecific's mouth (conspecific feeding task), 2) reaching-grasping and placing the sugar lump into the mouth-like aperture (body-shape placing task). The participants grasped the sugar lump using their right thumb and index finger (i.e. with a precision grasp). In both tasks, the participant was required to move with a natural velocity as during spontaneous movements and to put carefully the sugar lump into the mouth or the mouth-like aperture. Figure 1 shows the apparatus, stimuli, and tasks of the experiment. In the conspecific feeding task, the receiver's mouth was opened, before and during the trial. In all experiments her gaze was directed at a point just beyond the participants' left face. The receiver never came into eye contact with the giver in order to avoid that a mutual gaze interfered with the visual control of the execution of the sequence. The participants were requested to look at the opened mouth or the mouth-like aperture in front of them before starting the motor sequence; once the “GO” signal was given, they were free to look at the scene as during natural interactions with objects and people. The two tasks were executed in blocks of 8 trials with counterbalanced order across the participants.

#### Data recording

Movements of the participants' right hand were recorded using the 3D-optoelectronic SMART system (BTS Bioengineering, Milano, Italy). This system consists of six video cameras detecting infrared reflecting markers (spheres of 5-mm diameter) at a sampling rate of 120 Hz. Spatial resolution of the system is 0.3 mm. Recorded data were filtered using a linear smoothing low pass filter, i.e. a triangular filter where each value was the weighted mean computed over 5 samples (window duration: 33.3 ms).

We used three markers attached to the tip of the index finger, the thumb, and to the wrist of the participant's right hand. Other two markers were attached one to the upper and one to the lower lip of the human receiver, or in the case of the non-human receiver to the upper and lower edges of the mouth-like aperture, and were used as reference points. We analyzed the time course of the distance between the two markers placed on the two fingertips to study the grasp. The grasp time course starts with the hand in pinch position, and is constituted by a finger opening phase until a maximum (maximal finger aperture) followed by a phase of finger closing on the object [19]. We analyzed peak velocity of finger opening, and maximal finger aperture. The kinematics of the marker placed on the wrist was used to study the reaching and placing. We analyzed the following reach parameters: reach peak velocity, reach peak deceleration, and percentage of reach deceleration time (duration of the deceleration with respect to reach time). Peak velocity is a parameter related to the control of the first (acceleration) phase of reach, whereas percentage of deceleration time and peak deceleration are parameters related to the control of the second (deceleration) phase of reach. Percentage of deceleration time also takes into account the first phase of reach, whereas peak deceleration concerns the control of the second phase of reach only. The placing parameters analyzed were placing time, placing peak velocity and variability (SD) of placing end point along participants' Y and Z axes (YSD and ZSD), i.e. the variability on the receiver's coronal plane.

The methods for calculating the beginning and end of reach and grasp is described elsewhere [10]. The frame successive to the end of reach was considered to be the time of placing beginning. In order to determine the placing end we searched for the frames when, along the longitudinal, vertical and transverse axes of the participant, the displacement of the marker was smaller than 0.3 mm (spatial resolution of the system) with respect to the previous frame. The last frame was then selected as time of placing end.

#### Data analysis

ANOVAs were carried out on the mean values of the reaching-grasping and placing parameters. The within-subjects factor was task (conspecific feeding task vs body-shape placing task). The significance level was fixed at P<0.05. When the factor was significant, we also calculated the effect size [η^2^_p(artial)_].

### Results and Discussion

**Figure 2:**
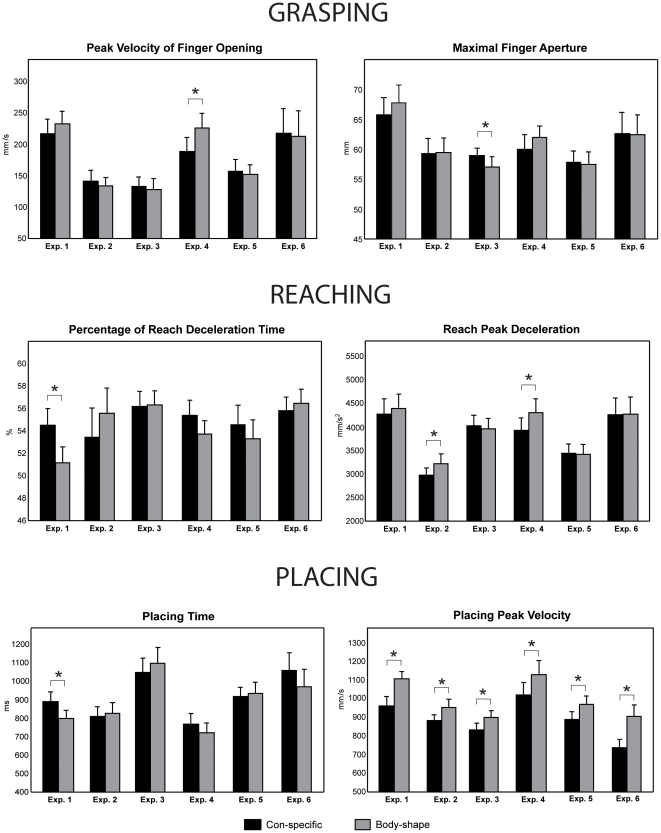
Mean values of kinematic parameters of grasping, reaching and placing analyzed in experiments 1–6. Bars are SE. Asterisk indicates significance in the ANOVAs.

As compared to the body-shape placing task, the conspecific feeding task showed an increase in percentage of reach deceleration time, and placing time, and a decrease in placing peak velocity (Table 1 and Fig. 2). Variabilty of placing end point on receiver's coronal plane was not affected by task (YSD F(1,11) = 0.16, n.s., ZSD F(1,11) = 0.19, n.s.).

**Table 1:** Results of the ANOVAs performed on the kinematic parameters collected in experiments 1–6.

		FACTOR TASK					
		Experiment 1	Experiment 2	Experiment 3	Experiment 4	Experiment 5	Experiment 6
**GRASPING**	**Peak Velocity of Finger Opening** (mm/s)	F(1,11) = 0.8, n.s.	F(1,11) = 0.5, n.s.	F(1,11) = 0.7, n.s.	F(1,11) = 7.1, p = 0.022, η^2^_p_ = 0.4	F(1,11) = 0.12, n.s.	F(1,11) = 0.15, n.s.
	**Maximal Finger Aperture** (mm)	F(1,11) = 3.6 p = 0.08	F(1,11) = 0.02, n.s.	F(1,11) = 6, p = 0.032; η^2^_p_ = 0.4	F(1,11) = 1.6, n.s.	F(1,11) = 0.10, n.s.	F(1,11) = 0.02, n.s.
**REACHING**	**Reach Peak Velocity** (mm/s)	F(1,11) = 0.3, n.s.	F(1,11) = 0.4, n.s.	F(1,11) = 0.5, n.s.	F(1,11) = 0.15, n.s.	F(1,11) = 0.01, n.s.	F(1,11) = 0.58, n.s.
	**Percentage of Reach Deceleration Time** (%)	F(1,11) = 6.7, p = 0.025; η^2^_p_ = 0.4	F(1,11) = 1.1, n.s.	F(1,11) = 0.02, n.s.	F(1,11) = 4, p = 0.07	F(1,11) = 1.35, n.s.	F(1,11) = 0.26, n.s.
	**Reach Peak Deceleration** (mm/s^2^)	F(1,11) = 0.9, n.s.	F(1,11) = 7.2, p = 0.021; η^2^_p_ = 0.4	F(1,11) = 0.12, n.s.	F(1,11) = 7.1, p = 0.021; η^2^_p_ = 0.4	F(1,11) = 0.09, n.s.	F(1,11) = 0.01, n.s.
**PLACING**	**Placing Time** (ms)	F(1,11) = 12.1, p = 0.005; η^2^_p_ = 0.5	F(1,11) = 0.1, n.s.	F(1,11) = 1.5, n.s	F(1,11) = 4.3, p = 0.06	F(1,11) = 0.18, n.s.	F(1,11) = 1.97, n.s.
	**Placing Peak Velocity** (mm/s)	F(1,11) = 33, p<0.001; η^2^_p_ = 0.7	F(1,11) = 5.8, p = 0.034; η^2^_p_ = 0.4	F(1,11) = 39.2, p<0.001; η^2^_p_ = 0 .8	F(1,11) = 25, p<0.001; η^2^_p_ = 0.7	F(1,11) = 9.1, p = 0.01; η^2^_p_ = 0.5	F(1,11) = 52.1, p<0.001; η^2^_p_ = 0.8

η^2^_p_: partial eta squared.

The slowing down of the final phase of reach, and of the whole placing, observed in the conspecific feeding task indicates an increasing accuracy in the control of all the sequence, due to an interaction with the entire receiver's body, rather than in the control of the final placing phase, due to an interaction with the receiver's mouth only (see the results concerning variability of placing end point). A first hypothesis explaining these results is that they are due to a social affordance implicitly demanding greater visual control of sequence execution. The social affordance could be activated by the social request to be fed (remember that the conspecific's mouth was opened before and during sequence execution). A second consistent hypothesis is that the hand trajectory was close to the conspecific's body (i.e. inside the peripersonal space, [14]); this could require greater accuracy in trajectory control in order to avoid touching the conspecific's (receiver's) body. These two hypotheses were tested in experiments 2 and 4 (first hypothesis) and 3 (second hypothesis).

## Experiment 2

In experiment 2 we dissociated the social request to be fed from the final intention of the sequence of reaching-grasping and placing; specifically, the action sequence was directed to a mouth-like aperture in a support placed either beside the conspecific's face (close-to-conspecific placing task) or the “face” of the body shape (close-to-body-shape placing task, Fig. 1). Before and during the conspecific placing task the conspecific's mouth was opened as in experiment 1. If the social request (i.e. the opened mouth) is responsible for increasing accuracy in sequence control because a social affordance is activated, we could find an effect on sequences directed to the conspecific even when they were unrelated to feeding.

### Methods

#### Participants

A new sample of twelve right-handed [18], naive volunteers (6 females and 6 males, age 20–23 yrs.) participated in the experiment.

#### Apparatus, stimuli and procedure

The same apparatus and stimuli as in experiment 1 were used, except that the final target of the sequence of actions was an ellipse-shaped slit resembling a human mouth (mouth-like aperture) whose size was approximately the same as that of the human receiver's mouth (when it was opened). The support of the mouth-like aperture was placed either beside the conspecific's right cheek or the corresponding position of the human body shape used in experiment 1 (Fig. 1). The participants were required to reach-grasp and place the sugar lump into the mouth-like aperture next the right conspecific's cheek (close-to-conspecific placing task) or the corresponding position of the human body-shape's (close-to-body-shape placing task). The conspecific's mouth was opened as in experiment 1. The remaining procedure was the same as in experiment 1.

#### Movement recording and data analysis

Movement recording and data analysis were the same as in experiment 1 except that two markers were attached to the upper and lower edges of the mouth-like aperture beside the conspecific's right cheek or the corresponding position of the human body shape. In the ANOVAs the within-subjects factor was task (close-to-conspecific placing task vs close-to-body-shape placing task).

### Results

Reach peak deceleration and placing peak velocity decreased in the close-to-conspecific placing task as compared to the close-to-body-shape placing task (Table 1 and Fig. 2). In sum, both the final reach and the placing were slowed down in the close-to-conspecific placing task.

## Experiment 3

In experiment 3 the participants executed the same two tasks (i.e. close-to-conspecific placing and close-to-body-shape placing); however, the position of the sugar lump was closer to the conspecific's body as compared to experiments 1 and 2. Differently from experiment 2, the conspecific's mouth was closed. If the peripersonal space is responsible for increasing accuracy during reaching and placing, we should find greater effect for hand trajectories closer to the conspecific's body.

### Methods

#### Participants

A new sample of twelve right-handed [18], naïve volunteers (7 females and 5 males, age 22–25 yrs.) participated in the experiment.

#### Apparatus and stimuli

The apparatus and stimuli were the same as those for experiment 2, except that the position of the sugar lump was closer (12 cm instead of 22 cm) to the conspecific and to human body-shape facing the participants, i.e. it was farther (26 cm distant) from the SP (Fig. 1). Moreover, the conspecific's mouth remained closed before and during the trial. Correspondingly, a wooden plate covered the mouth-like aperture.

#### Procedure, movement recording and data analysis

The procedure, movement recording and data analysis were the same as in experiment 2.

### Results and Discussion

Maximal finger aperture increased and placing peak velocity decreased in the close-to-conspecific placing task as compared to the close-to-body-shape placing task (Table 1 and Fig. 2).

The closeness of hand trajectory to the conspecific's body induced an increase in maximal finger aperture. Larger maximal finger apertures allow compensation for an increase in uncertainty in the hand trajectory control [20]. When the fingers moved inside the peripersonal space during the final reaching-grasping the salience of the context probably increased. Thus, the attention of the agent focused on the conspecific's body to a greater extent causing uncertainty and less control of hand trajectory.

By comparing the results of experiment 2 with those of experiment 3 we deduce that the social request to be fed (i.e. the conspecific's mouth aperture) is sufficient to activate a social affordance even when the giver does not actually interact with a present conspecific, and, in particular, with her mouth. This slows down the second phase of reach of the sequence not actually finalized to feed. In contrast, the closeness of hand trajectory to the conspecific's body has a minor effect on the reach kinematics, whereas it greatly affects the grasp (see above).

## Experiment 4

The results of experiments 1 and 2 suggest that the effect of the social request (and of the corresponding social affordance) was stronger in experiment 1 than in experiment 2, i.e. when the social intention and social request were congruent. Indeed, in experiment 1, the social affordance affected the percentage of deceleration time, which also takes into account the first (acceleration) phase of reach, whereas in experiment 2, it affected reach peak deceleration which concerns the second (deceleration) phase of reach only.

In experiment 2 a direct interaction with the conspecific was precluded and the hand trajectory during the placing directed away from the conspecific's face. On the basis of these data we hypothesized that a greater effect of the social request could be found during direct interactions with the conspecific, even if they are not finalized to feed, and when hand trajectories were closer to the mouth. In order to test this hypothesis, in experiment 4 participants reached-grasped the sugar lump and with this in their hand they touched the conspecific's forehead (conspecific touching task) or the “forehead” of the body-shape (body-shape touching task). The conspecific's mouth was opened as in experiment 2 and the hand trajectory was closer to the conspecific's body as in experiment 3.

### Methods

#### Participants

A new sample of twelve right-handed [18], naïve volunteers (8 females and 4 males, age 23–25 yrs.) participated in the experiment.

#### Apparatus and stimuli

The apparatus and stimuli were the same as those of experiment 1. However, differently from experiment 1, the position of the sugar lump was the same as that in experiment 3.

#### Procedure, movement recording and data analysis

The participants reached-grasped the sugar lump and with this in their thumb and index finger touched the conspecific's forehead (conspecific touching task) or the “forehead” of the body-shape (body-shape touching task) (Fig. 1). The conspecific's mouth was opened as in experiment 2. The remaining procedure and movement recording were the same as in experiment 1 except that one reference marker was attached to the forehead of the human or non-human receiver, in addition to the three markers attached to the thumb, index finger, and wrist of the participant. In the ANOVAs the within-subjects factor was task (conspecific touching task vs body-shape touching task).

### Results and Discussion

Peak velocity of finger opening, and reach peak deceleration decreased, whereas percentage of reach deceleration time showed a trend to increase, in the conspecific touching task as compared to the body-shape touching task (Table 1 and Fig. 2). Placing peak velocity decreased and placing time showed a trend to increase in the conspecific touching task (Table 1 and Fig. 2).

A direct interaction with the conspecific and a hand trajectory closer to the mouth increased the effect (i.e. the accuracy demand) of the social request to be fed and of the corresponding social affordance on a sequence not finalized to feed. The social affordance also affected the grasp since peak velocity of finger opening decreased. Consequently, the effect of the hand trajectory closeness to the conspecific's body found in experiment 3 (i.e. the increase in maximal finger aperture) was removed by the lower velocity of finger opening. Moreover, the results of both experiment 2 and 4 confirm that the social request affects the control of all the sequence rather than the final placing phase. In fact, the givers did not actually interact with the receiver's mouth. This was suggested in experiment 1by the results concerning variability of the placing end point.

## Experiment 5

The results of experiment 4 do not exclude that other factors inherent in the conspecific's face are responsible for effects on hand movements finalized to touch the conspecific's forehead; for example, the gaze of the conspecific. Indeed, it is well known that the conspecific's gaze can be a signal in order to initiate a communication ([15]; see also [16], concerning the structures in the social brain activated by the “eye contact effect”). Moreover, during interactions it can be a signal to make a social request effective and, consequently, to activate a social affordance. This hypothesis was tested in experiment 5, in which the same sequence of actions as in experiment 4 was directed to a blindfolded conspecific or a “blindfolded” body-shape.

### Methods

#### Participants

A new sample of twelve right-handed [18], naïve volunteers (6 females and 6 males, age 24–26 yrs.) participated in the experiment.

#### Apparatus and stimuli

The apparatus and stimuli were the same as those of experiment 4, except that the conspecific and the human-body shape were blindfolded (Fig. 1).

#### Procedure, movement recording and data analysis

Procedure, movement recording and data analysis were the same as in experiment 4. In addition, a second series of ANOVAs was carried out on mean values of the reaching-grasping and placing parameters of experiments 4 and 5. They included the within-subjects factor task (conspecific touching task vs body-shape touching task) and the between-subjects factor experiment (4 vs 5). In all analyses post-hoc comparisons were performed using the Newman-Keuls procedure. The significance level was fixed at P<0.05.When the factor was significant, we also calculated the effect size [η^2^_p(artial)_].

### Results and Discussion

In the first series of ANOVAs no parameter was affected by factor task except placing peak velocity, which decreased in the conspecific touching task (Table 1 and Fig. 2). Concerning the second series of ANOVAs, peak velocity of finger opening decreased when the participants interacted with the conspecific, but only in experiment 4, i.e. when the receiver's gaze was available (interaction between task and experiment, (F(1, 22) = 5.0, p<0.05, η^2^_p_ = 0.2, post-hoc test p<0.05, Fig. 2). Similarly, reach peak deceleration decreased in conspecific touching task, but in experiment 4 only (interaction between task and experiment, F(1, 22) = 5.9, p<0.05, η^2^_p_ = 0.2, post-hoc test p<0.05, Fig. 2). In contrast, percentage of reach deceleration time increased in conspecific touching task in both the experiments (factor task: F(1, 22) = 4.5, p<0.05, η^2^_p_ = 0.2, Fig. 2). Placing peak velocity decreased in conspecific touching task (F(1, 22) = 29.8, p<0.001, η^2^_p_ = 0.6, Fig. 2).

The results of experiment 5 confirm that the gaze of the human receiver plays a role in activating a social affordance. This is mainly shown by the finding that reach peak deceleration and peak velocity of finger opening decreased in conspecific touching task only when the receiver's gaze was available. However, percentage of reach deceleration time increased even when the receiver's gaze was not available. This result may depend on an effect of the social intention of touching in experiments 4 and 5. Since the giver's social intention of touching was not coupled with any social request, it was less affected by the preclusion of the receiver's gaze. Indeed, we propose that the receiver's gaze makes effective a social request (e.g. to be fed) for the activation of a social affordance.

## Experiment 6

The results of experiment 5 do not completely solve the problem of whether the receiver's gaze plays a primary role in making the social request to be fed effective to activate a social affordance. In other words, can the social intention of feeding activate a social affordance independently of the effects of the receiver's gaze on the social request? Experiment 6 aimed at solving this problem: we compared a conspecific feeding task with a body-shape placing task, as in experiment 1, during which the (human and non-human) receiver was blindfolded. If a social affordance is only activated by a social request coupled with the gaze of the receiver, we should find no effect of the conspecific feeding task on the reach.

### Methods

#### Participants

A new sample of twelve right-handed [18], naïve volunteers (8 females and 4 males, age 23–27 yrs.) participated in the experiment.

#### Apparatus and stimuli

The apparatus and stimuli were the same as those of experiment 5.

#### Procedure, movement recording and data analysis

The participants executed a conspecific feeding task and a body-shape placing task as in experiment 1, during which the conspecific and the human-body shape were blindfolded (Fig. 1). Movement recording and data analysis were the same as in experiment 1. In a second series of ANOVAs we compared experiment 6 with experiment 1; the within-subjects factor was task (conspecific feeding task vs a body-shape placing task) and the between-subjects factor was experiment (1 vs 6).

### Results and Discussion

In the first series of ANOVAs no parameter was affected by the factor task except placing peak velocity, which decreased in the conspecific feeding task (Table 1 and Fig. 2). In the second series of ANOVAs percentage of reach deceleration time was affected by the interaction between factors task and experiment (F(1, 22) = 4.8, p<0.05, η^2^_p_ = 0.2). This parameter increased during the conspecific feeding task as compared to body-shape placing task, in experiment 1 only (post-hoc test, p<0.05, Fig. 2). Placing time increased (F(1, 22) = 4.2, p = 0.05, η^2^_p_ = 0.2) and placing peak velocity decreased (F(1, 22) = 83.04, p<0.001, η^2^_p_ = 0.8) in conspecific feeding task (Fig. 2). Factor experiment affected placing time and placing peak velocity (F(1, 22) = 6.6; p<0.05, η^2^_p_ = 0.2; F(1, 22) = 9.9, p<0.01, η^2^_p_ = 0.3): placing time increased and placing peak velocity decreased in experiment 6 (Fig. 2).

The receiver's gaze makes the social request to be fed (mouth aperture) effective at activating a social affordance. In other words, the social intention of feeding alone is unable to activate a social affordance if it is not coupled with an effective social request.

## General Discussion

In experiment 1, we compared the social intention of feeding a conspecific with the intention of placing a piece of food into a mouth-like aperture in a human body shape: both the intentions guided the same action sequence constituted by reaching-grasping and placing. The feeding intention increased the accuracy requirement of the overall sequence: indeed, the reaching as well as the placing slowed down. These results confirm the data of the study by Ferri and colleagues [13]. The increasing accuracy demand due to the social intention affected the action of reaching in line also with kinematic data showing that actions in a chain are related to each other [4], [6]–[8]. The increasing accuracy demand during the control of reaching and placing might depend on the final contact with the receiver's mouth because the participants may want to avoid touching the receiver's lips. The results concerning variability of placing end point in experiment 1 rule out this possibility. Moreover, the results of experiment 6 showed that the final contact with the receiver's mouth induced a decrease in placing peak velocity only, as for the case in experiment 3 when the final contact was with a mouth-like aperture (placed beside the conspecific's face). In addition, the results of experiment 3 exclude that the increasing demand of accuracy observed in reaching and placing was due to the closeness of the hand trajectory to the conspecific's body, which the participants might avoid touching. In fact, it was the grasp, rather than the reach, which was influenced by closer trajectories. However, in all the experiments the presence of the conspecific induced a decrease in placing peak velocity even when the sequence was directed to another final target (experiments 2 and 3). It is possible that, when planning the sequence, the maximal velocity of the placing (during which the hand trajectory was closer to the conspecific's body) was reduced in order to facilitate a quick reaction in response to unexpected movements of the conspecific. A similar explanation may be offered for the trend of slowing down of the placing when the receiver was blindfolded. In fact, since the possibility of trunk oscillations was higher when vision was precluded to the receiver, the givers might plan lower placing velocities in order to quickly react to possible trunk oscillations of the receiver.

Thus, we propose that a social affordance is activated when feeding a conspecific. The social request, i.e. the conspecific's mouth aperture signaling a request to be fed, was a prerequisite for activating a social affordance. The social affordance activated by the social request to be fed influenced both the reaching and placing even when this sequence was not finalized to feed (experiments 2 and 4), i.e. in the case of not actual interaction with the conspecific's mouth. This is in agreement with the data by Sartori and colleagues [21]. These authors studied the interference effects of a sudden presentation of a social request, i.e. the hand opening expressing “give-me-the-object”, on the execution of a sequence directed to an inanimate final target. The social request interfered with the actual sequence by inducing a partial deviation of the hand trajectory towards the conspecific. Conversely, we studied the effects of the social request to be fed (i.e. the mouth aperture) on planning of sequences unrelated to feeding directed to inanimate (experiment 2) and animate (experiment 4) final targets. The social request was presented well in advance of sequence beginning, and the time of presentation was sufficient to remove eventual transitory effects on sequence control. The social request affected the planning of the sequence because the corresponding social affordance changed movement parameterization (i.e. modified kinematic landmarks). In other words, the social affordance was included in the planning. A possibility explaining this effect is that in experiment 2 the actual sequence resembled a feeding and its initial part was directed towards the conspecific's body. Conversely, in experiment 4 placing the sugar lump into the conspecific's mouth, as required by the social request, could be more natural than touching the conspecific's forehead at the end of the actual sequence as required to the giver. Moreover, the actual sequence was not preceded by any related social request. Summing up, the congruence between the social request and the possible intentions guiding the sequence was sufficient to include the social affordance into the planning of the actual sequence.

The results of experiments 5 and 6 suggest that the conspecific's gaze is coupled with specific social requests (for example, mouth aperture requiring to be fed). This is in agreement with the data by Sartori, Becchio, Bulgheroni, & Castiello [21]. We found that the specific social request was ineffective if the conspecific's gaze was precluded to the agent. On the basis of these results, we propose that the conspecific's gaze expresses a cue [15] to indicate that the successive signalling (i.e. the request) is deliberate. The production of the signal indicates two things: first, that the person wishes to activate an interaction; second, that the successive signal (a request gesture, in the present study the mouth aperture) coupled with the gaze is relevant to the interest of the receiver [22]. When an interaction is required, this signal activates a social affordance. This, in the present study, induces an increase in movement accuracy just because the receiver implicitly requires a visual control on sequence execution. This can occur even in the case of no direct eye contact with the agent, as we found in the present study. Kilner, Marchant, and Frith [23], using magnetoencephalography recorded cortical activity of humans observing videos showing movements of an actor. The attenuation of the oscillatory activity during movement observation depended on whether the actor was facing towards or away from the observer. Specifically, the authors found attenuation in the pattern elicited by movement observation only when the actor was facing towards the observer. This result suggests that the effects of gesture observation are modulated by the relationships between observer and actor. In other words, even more simple automatic imitations as compared to the more complex interactions require that the conspecific gaze is available in order to be activated. In neural terms, candidates for the coding of related-to-gaze intentionality are posterior STS (Superior Temporal Sulcus) and medial prefrontal cortex (for reviews see [16], [24]). This proposal is corroborated by results of single neuron recording studies in STS of monkey cortex [25].

## References

[1] Fitts PM (1954). The information capacity of the human motor system in controlling the amplitude of movement.. Journal of Experimental Psychology: Human Perception and Performance.

[2] Bootsma RJ, Marteniuk RG, MacKenzie CL, Zaal FT (1994). The speed-accuracy trade-off in manual prehension: effects of movement amplitude, object size and object width on kinematic characteristics.. Exp Brain Res.

[3] Gentilucci M, Castiello U, Corradini ML, Scarpa M, Umilta C (1991). Influence of different types of grasping on the transport component of prehension movements.. Neuropsychologia.

[4] Marteniuk RG, MacKenzie CL, Jeannerod M, Athenes S, Dugas C (1987). Constraints on human arm movement trajectories.. Can J Psychol.

[5] Mon-Williams M, McIntosh RD (2000). A test between two hypotheses and a possible third way for the control of prehension.. Exp Brain Res.

[6] Johnson-Frey S, McCarty M, Keen R (2004). Reaching beyond spatial perception: effects of intended future actions on visually-guided prehension.. Visual Cognition.

[7] Gentilucci M, Negrotti A, Gangitano M (1997). Planning an action.. Exp Brain Res.

[8] Rosenbaum D, Jorgensen M (1992). Planning macroscopic aspects of manual control.. Human Movement Science.

[9] Fogassi L, Ferrari PF, Gesierich B, Rozzi S, Chersi F (2005). Parietal Lobe: From Action Organization to Intention Understanding.. Science.

[10] Barbieri F, Buonocore A, Bernardis P, Volta RD, Gentilucci M (2007). On the relations between affordance and representation of the agent's effector.. Exp Brain Res.

[11] Loveland K (1991). Social affordances and interaction II: autism and the affordances of the human environment.. Ecological psychology.

[12] Becchio C, Sartori L, Bulgheroni M, Castiello U (2008). The case of Dr. Jekyll and Mr. Hyde: a kinematic study on social intention.. Conscious Cogn.

[13] Ferri F, Campione GC, Dalla Volta R, Gianelli C, Gentilucci M (2010). To me or to you? When the self is advantaged.. Exp Brain Res.

[14] Gentilucci M, Scandolara C, Pigarev IN, Rizzolatti G (1983). Visual responses in the postarcuate cortex (area 6) of the monkey that are independent of eye position.. Exp Brain Res.

[15] Frith C (2009). Role of facial expressions in social interactions.. Philos Trans R Soc Lond B Biol Sci.

[16] Senju A, Johnson MH (2009). The eye contact effect: mechanisms and development.. Trends Cogn Sci.

[17] Sartori L, Becchio C, Bara BG, Castiello U (2009). Does the intention to communicate affect action kinematics?. Consciousness and Cognition.

[18] Oldfield RC (1971). The assessment and analysis of handedness: the Edinburgh inventory.. Neuropsychologia.

[19] Jeannerod M (1988). The Neural and Behavioural Organization of Goal-directed Movements.

[20] Chieffi S, Gentilucci M (1993). Coordination between the transport and the grasp components during prehension movements.. Exp Brain Res.

[21] Sartori L, Becchio C, Bulgheroni M, Castiello U (2009). Modulation of the action control system by social intention: unexpected social requests override preplanned action.. J Exp Psychol Hum Percept Perform.

[22] Sperber D, Wilson D (1995). Relevance: communication and cognition.

[23] Kilner JM, Marchant JL, Frith CD (2006). Modulation of the mirror system by social relevance.. Soc Cogn Affect Neurosci.

[24] Puce A, Perrett D (2003). Electrophysiology and brain imaging of biological motion.. Philosophical Transactions of the Royal Society Biological Sciences.

[25] Oram M, Perrett D (1993). Integration of form and motion in the anterior superior polysensory area (STPa) in the macaque monkey.. Journal of Neurophysiology.

